# Recombinant Klotho protein enhances cholesterol efflux of THP-1 macrophage-derived foam cells via suppressing Wnt/β-catenin signaling pathway

**DOI:** 10.1186/s12872-020-01400-9

**Published:** 2020-03-05

**Authors:** Wei Liu, Xiujuan Chen, Min Wu, Lin Li, Jiani Liu, Jing Shi, Tian Hong

**Affiliations:** Department of Gerontology, the Central Hospital of Wuhan, Tongji Medical College, Huazhong University of Science and Technology, Wuhan, 430014 China

**Keywords:** Recombinant Klotho protein, Cholesterol efflux, THP-1 macrophage-derived foam cells, Wnt/β-catenin signaling pathway, Atherosclerosis

## Abstract

**Background:**

Atherosclerosis (AS) is the basis of cardiovascular diseases, characterized by chronic inflammatory and lipid metabolism disorders. Although the anti-inflammatory effect of Klotho in AS has been clearly shown, its lipid-lowering effect is unclear. In this study, we examined the effects of recombinant Klotho (Re-KL) protein on lipid accumulation in foam cells.

**Methods:**

THP-1 cells were exposed to 100 nM phorbol myristate acetate for 24 h and then to oxidized low-density lipoprotein (ox-LDL; 80 mg/mL) to induce foam cell formation. Subsequently, the foam cells were incubated with Re-KL and/or DKK1, an inhibitor of the Wnt/β-catenin pathway.

**Results:**

Oil red O staining and cholesterol intake assay revealed that the foam cell model was constructed successfully. Pre-treatment of the foam cells with Re-KL decreased total cholesterol level, up-regulated the expression of ATP binding cassette transporter A1 (ABCA1) and G1 (ABCG1), and down-regulated the expression of acyl coenzyme a-cholesterol acyltransferase 1 (ACAT1) and members of the scavenger family (SR-A1 and CD36). In addition, the expression of Wnt/β-catenin pathway-related proteins in foam cells was significantly decreased by the stimulus of Re-KL. Interestingly, the effect of Re-KL was similar to that of DKK1 on foam cells.

**Conclusions:**

The Re-KL-induced up-regulation of reverse cholesterol transport capacity promotes cholesterol efflux and reduces lipid accumulation by suppressing the Wnt/β-catenin pathway in foam cells.

## Background

Atherosclerosis (AS) is characterized by chronic inflammation and disorders of lipid metabolism, and is one of the leading causes of human morbidity and death [1]. Previous studies from numerous researchers have recognized that the accumulation of excess cholesterol as a crucial event in the occurrence and development of AS [2, 3]. Therefore, preventing or reversing cholesterol accumulation may be effective protective strategies against AS. Throughout the years multiple therapeutic approaches in the cardiovascular field have been approved. Among them, statins and anti-hypertensive agents have shown the ability to modify immunological and inflammatory responses in parallel to their principal effects as cholesterol-lowering or blood pressure reduction agents [4, 5]. Moreover, new drugs targeting specific targets in the inflammatory cascade are also being used in clinical practice [6]. Therefore, based on the complexity of the pathogenesis of AS, it is of great significance to develop more effective and targeted therapeutic drugs.

A growing body of evidence suggests that high density lipoprotein (HDL) plays an important role in the removal of cholesterol from atherosclerotic plaques and the transport of excess cholesterol back to the liver, and this is one of the main mechanisms to prevent the occurrence of AS, which called reverse cholesterol transport (RCT) [7–9]. ATP binding cassettes (ABC) A1 and G1 are key genes in RCT process [10, 11]. ABCA1 has been reported to play an important role in the prevention of AS by facilitating cholesterol efflux and decreasing cholesterol accumulation in macrophages [10, 12]. Similar to ABCA1, ABCG1 plays a synergistic role in mediating cholesterol efflux to HDL and further exerts anti-AS effects [13]. However, acyl coenzyme a-cholesterol acyltransferase (ACAT) plays an important role in the absorption, transportation, and storage of cholesterol and is one of the key proteins that regulate the balance of cholesterol metabolism in the body [8, 14]. In addition, during the formation of foam cells, members of the scavenger receptor family such as scavenger receptor-A1 (SR-A1) and CD36 mediate the sustained uptake of oxidized low density lipoprotein (ox-LDL) [15]. Therefore, the dynamic change of these indicators is an important factor in measuring the physiological function of foam cells.

Klotho is an anti-aging factor involved in different biological processes, mainly expressed in the kidney, parathyroid gland and choroid plexus, but recent studies have shown that human vascular tissue also express this protein [16, 17]. A clinical study has newly identified lower serum Klotho level as a predictor of AS in patients [18]. Inflammation is critical for the development and progression of AS [19]. Incubation with Klotho inhibits monocyte adhesion and reduces expression of adhesion molecules in human umbilical vein endothelial cells (HUVEC) [20]. Although the anti-inflammatory effect of Klotho in AS has been very clearly demonstrated, its lipid-lowering effect has not yet been elucidated.

In the present study, we hypothesized that the formation of foam cells in AS may induce a reduction in Klotho. Thus, this work investigated the effect of Klotho on cholesterol efflux and key genes related to cholesterol metabolism in terms of mRNA and protein expression in ox-LDL-stimulated THP-1 macrophage-derived foam cells. In addition, the mechanism by which Klotho induces these effects was also investigated.

## Methods

### Materials and reagents

RPMI-1640 medium (SH30027), fetal bovine serum (FBS, SH30088.03HI) and bovine serum albumin (BSA, SH3057401) were acquired from GE Healthcare Life Sciences HyClone (Logan, UT, USA); Phorbol myristate acetate (PMA; ICA1042) was purchased from Gene Operation (Ann Arbor, Michigan, USA); ox-LDL (H7950) was purchased from Solarbio (Beijing, China); Recombinant human Dickkopf-related protein 1 (O94907) was purchased from R&D systems China Co.,Ltd. (Shanghai, China); DAPI (D9542) were purchased from Sigma-Aldrich (St Louis, MO, USA); Total cholesterol assay kit (F002–1), Total protein extraction kit (W034), Nuclear protein extraction kit (W037), Membrane protein and cytoplasmic protein extraction kit (W036) and The total protein assay kit (BCA method, A045–4) were purchased from Jiancheng Bioengineering Institute (Nanjing, China); Cholesterol ester assay kit (XFC1377) was purchased from Xinfan biotechnology co., LTD (Shanghai, China); TRIzol reagent (15596026), cDNA synthesis kit (N8080234) and SYBR Green PCR Master Mix (4312704) were purchased from Invitrogen/ThermoFisher Scientific, Inc. (Waltham, MA, USA); Recombinant human Klotho protein (ab84072), anti-c-Myc (ab32072), anti-beta Catenin (ab32572), anti-Cyclin D1 (ab134175), anti-SRA1 (ab183725), anti-CD36 (ab133625), anti-ACAT1 (ab168342), anti-ABCA1 (ab7360), anti-ABCG1 (ab52617), anti-beta Actin (ab8227), anti-GAPDH (ab37168), anti-Histone H3 (ab8580) primary antibodies and HRP (ab6721)/FITC (ab6717)-conjugated anti-rabbit secondary antibodies were purchased from Abcam (Cambridge, UK).

### Construction and evaluation of foam cell model

Human THP-1 cells (TIB-202) were purchased from American Type Culture Collection (ATCC, Manassas, VA, USA) and cultured in RPMI-1640 (10% FBS, 20 μg/mL streptomycin and 20 IU/mL penicillin) at 37 °C in a humidified atmosphere containing 5% CO_2_, and then treated with 100 nM PMA for 24 h. Subsequently, the medium was replaced with fresh medium and the cells were incubated with 80 mg/mL ox-LDL for 48 h to establish the model of THP-1 macrophage-derived foam cells. The cultured foam cell model was evaluated by oil red O staining and cholesterol intake assay as previously described [21].

### Determination of the expression of Klotho and key molecules related to Wnt/β-catenin signaling pathway in foam cell model

Under the premise of constructing the foam cell model successfully, we investigated the correlation between foam cell formation and the expression of Klotho and key molecules related to the Wnt/β-catenin signaling pathway. THP-1 macrophages without ox-LDL induction were used as the control group. Quantitative reverse-transcription polymerase chain reaction (qRT-PCR) and western blot were performed to detect the expression of Klotho at the mRNA and protein levels, respectively. Total cellular protein was extracted by nucleoplasm isolation, and the localization and content of each key gene related to the Wnt/β-catenin signaling pathway (β-catenin, c-Myc, and cyclin D1) were evaluated. In addition, immunofluorescence staining was conducted to assess the intracellular expression and distribution of β-catenin.

### Effect of different concentrations of recombinant Klotho on the activity of foam cells (MTT assay)

The THP-1 macrophage-derived foam cells (1.0 × 10^4^ cells/mL) were seeded in 96-well microtiter plates. The cells were then incubated with various concentrations of recombinant Klotho (25 ng/mL, 50 ng/mL, 100 ng/mL and 200 ng/mL) for 24 h or 50 ng/mL recombinant Klotho protein for different periods of time (6 h, 12 h, 24 h and 48 h). Subsequently, 10 μL MTT solution was added to each well, followed by incubation for 4 h at 37 °C. The absorbance was measured at the 490 nm using iMark microplate reader (Bio-Rad, California, USA). Cell viability was calculated as follows: cell viability (%) = [(treated group A value - untreated control A value)] / [(control group A value - untreated control A value)] × 100%.

### Effect of recombinant Klotho protein on cholesterol metabolism in foam cells

Cell experiments were divided into 3 groups: control group (no ox-LDL induced THP-1 macrophages); model group (THP-1 macrophage-derived foam cells) and treatment group (foam cells+ 50 ng/mL recombinant Klotho). After 24 h of co-culture, oil red O staining, total cholesterol and cholesterol ester assay were performed. qRT-PCR and western blot were conducted to measure the expression of key genes in cholesterol metabolism (SR-A1, CD36, ACAT1, ABCA1 and ABCG1) at mRNA and protoien levels, respectively. Total cellular protein was extracted by nucleoplasm isolation, and the localization and content of each key gene related to the Wnt/β-catenin signaling pathway (β-catenin, c-Myc, and cyclin D1) were evaluated. In addition, immunofluorescence staining was performed to assess the intracellular expression and distribution of β-catenin.

### The correlation between recombinant Klotho and Wnt/β-catenin signaling pathway in the biological function of foam cells

To investigate the specific mechanism of recombinant Klotho acting on foam cells, we selected recombinant human Dickkopf-related protein 1, DKK1, an inhibitor of the Wnt/β-catenin signal pathway, for joint studies. First, THP-1 macrophage-derived foam cells (1.0 × 10^4^ cells/mL) were seeded in 96-well microtiter plates. The cells were then incubated with various concentrations of DKK1 (100 ng/mL, 200 ng/mL, 400 ng/mL, and 800 ng/mL) for 24 h. The expression of β-catenin protien was used to evaluate the optimal concentration of DKK1 for foam cells. Then, the cell experiments were divided into 5 groups: control group (no ox-LDL induced THP-1 macrophages); model group (THP-1 macrophage-derived foam cells), treatment group 1 (foam cells + 50 ng/mL recombinant Klotho), treatment group 2 (foam cells + 400 ng/mL DKK1) and treatment group 3 (foam cells + 50 ng/mL recombinant Klotho + 400 ng/mL DKK1). After 24 h of co-culture, oil red O staining and total cholesterol assay were performed to detect the cholesterol efflux. qRT-PCR and western blot were conducted to evaluate the expression of key genes in cholesterol metabolism (SR-A1, CD36, ACAT1, ABCA1, and ABCG1) at the mRNA and protein levels, respectively.

### qRT-PCR

For qRT-PCR, total RNA was extracted from cells in each group using Trizol and reverse transcribed into cDNA using the cDNA synthesis kit according to the manufacturer’s instructions. Then, qRT-PCR was performed in a 20-μL reaction mixture containing SYBR Green PCR Master Mix, cDNA, and each primer at 0.2 mmol/L at 95 °C for 10 min, 40 cycles at 95 °C for 10 s, and 60 °C for 45 s. Data was collected using the QuantStudio™ 6 Flex Real-Time PCR System (Applied Biosystems, CA, USA). The relative amount of each gene was normalized to the housekeeping gene β-actin. Relative quantities were analyzed using the 2^-ΔΔCt^ method. Primer sequences are shown in Table 1.

**Table 1:** Primer sequences

Gene names	Forward sequence (5′-3′)	Reverse sequence (5′-3′)
Klotho	ACCAAGAAGAGGAAATC	TACCCAGAGGGAGAATC
SR-A1	CAGTGGGCTGGAGGAAA	ATGAGGGAGCGGTGGAT
CD36	ATTTCCACCTTTTGTTGA	ATAGTTGTCTGGGTTTTC
ACAT1	GCTGCTCTGGTTCTCAT	GGCTTCATTTACTTCCC
ABCA1	GAGGTTGCTGCTGTGGA	GGCATGGCTTTATTTGG
ABCG1	CAAGTCGGTGTGTGTCT	GCTTCCGTGAGGTTATT
β-Actin	ACACTGTGCCCATCTACG	TGTCACGCACGATTTCC

### Protein extraction and western blot analysis

The total proteins, nuclear proteins, and cytoplasmic proteins of each group were extracted using the corresponding extraction kits and quantified by the total protein assay kit (BCA method). With that, whole proteins were electrophoresed in 12% polyacrylamide gels [22]. The separated proteins were transferred to polyvinylidene difluoride membranes. Non-specific binding was blocked with 5% skimmed milk for 2 h at 37 °C. Then, the membranes were incubated with primary antibodies (Klotho, 1:1000; β-catenin, 1:10000; c-Myc, 1:10000; Cyclin D1, 1:10000; SR-A1, 1:5000; CD36, 1:5000; ACAT1, 1:1000; ATCA1, 1:1000; ABCG1, 1:5000) at 4 °C overnight. The GAPDH (1:1000), β-Actin (1:1000) and Histone H3 (1:1000) antibodies were used as controls. Next, the membranes were incubated with HRP-conjugated goat anti rabbit secondary antibody (1:10000) for 1 h at room temperature. At last, images were obtained from multifunctional Gel Imaging System (Image Quant LAS 500, General Electric, Fairfield, CT, USA), and the gray value of each band was quantified and analyzed using IQTL 8.1 software [23].

### Immunofluorescence staining

After the cell experiment, the culture supernatant was removed and the cells were washed softly with ice cold PBS, fixed with 4% paraformaldehyde, permeabilized in 0.1% Triton X-100 for 5 min, and washed with cold PBS twice. Then fixed cells were blocked with PBS containing 3% BSA for 1 h at room temperature. The cells were then incubated with the indicated primary antibodies overnight followed by incubation with FITC-labeled secondary antibody. Finally, the cell nuclei were stained with DAPI (1:800) and examined under a C2 laser scanning confocal microscope (Nikon, Japan). The Nikon NIS Elements AR imaging system was used to collect relevant information.

### Statistical analyses

All values are presented as the means ± SD. The *t* test was used to compare differences between two groups. The One-way ANOVA followed by the Tukey’s post hoc test was used to compare differences between multiple groups (more than 2) using SPSS 19.0 software (IBM Corp., Armonk, NY, USA). *P* < 0.05 was considered as a statistically significant difference.

## Results

### THP-1 macrophage-derived foam cell model

**Fig. 1:**
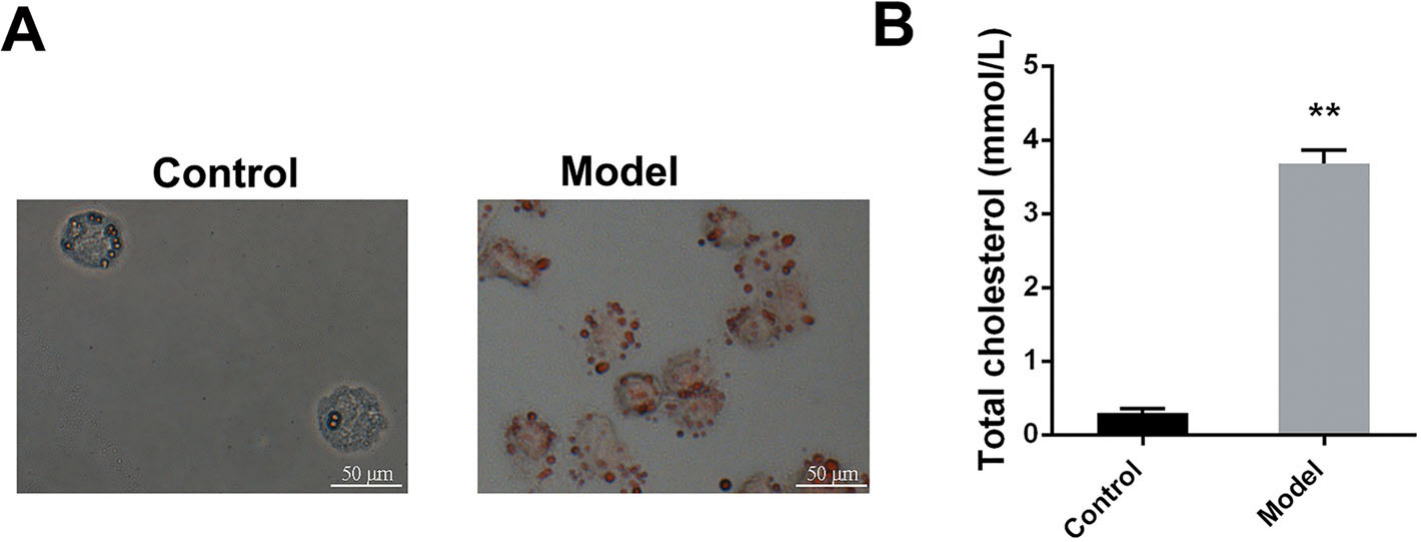
Construction and evaluation of foam cell model. (**a**) Oil red O staining (scale bar = 50 μm); (**b**) Total cholesterol level assay. ^**^P < 0.01 vs. control group

As shown in Fig. 1, compared with the control group (no ox-LDL induced THP-1 macrophages), the number of red lipid droplets engulfed by THP-1 macrophages increased significantly after ox-LDL induction (oil red O staining, Fig. 1a). After 48 h of co-incubation, the total intracellular cholesterol level in the THP-1 macrophages induced by ox-LDL increased significantly compared to that in thecontrol group (*P* < 0.01, Fig. 1b). This result suggests that we have successfully constructed the foam cell model.

### Expression of Klotho and key molecules related to Wnt/β-catenin signaling pathway in foam cell model

**Fig. 2:**
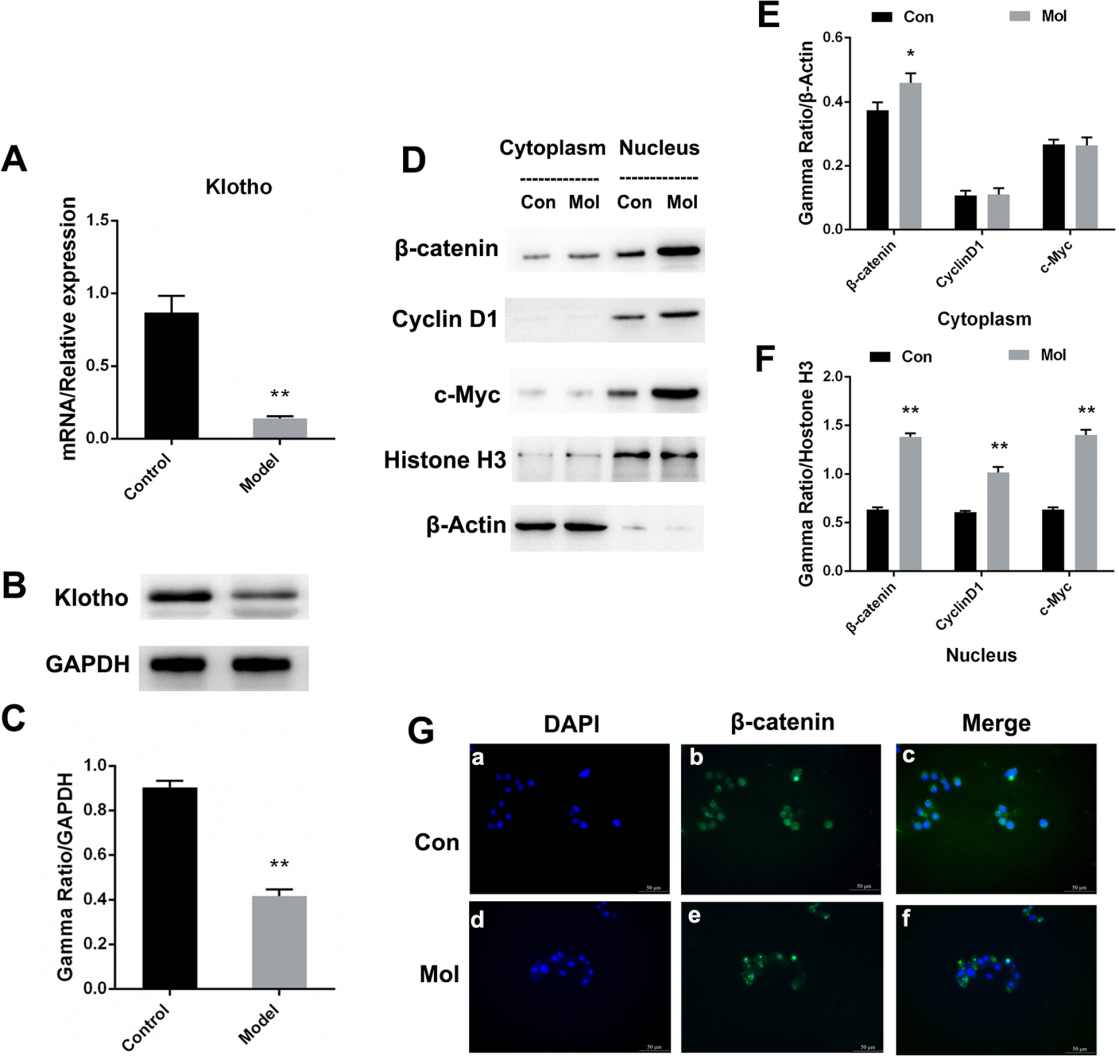
Expression of Klotho and key molecules related to the Wnt/β-catenin signaling pathway in foam cell model. **a/b/c** mRNA and protein expression of Klotho in control and model group; **d/e/f** Expression of key molecules related to Wnt/β-catenin signaling pathway; **g** Immunofluorescence staining of β-catenin (scale bar = 50 μm); **a/d** DAPI staining (blue); **b/e** β-catenin staining (green); **c/f** Merge; Con, Control group; Mol, Model group. ^*^P < 0.05 and ^**^P < 0.01 vs. control group

After building the foam cell model successfully, we detected the expression levels of Klotho and key molecules related to Wnt/β-catenin signaling pathway in foam cell model. As shown in the results of qRT-PCR, the mRNA level of Klotho in foam cells were significantly lower than that in the control group (*P* < 0.01, Fig. 2a). Similar to the qRT-PCR testing result, the protein level of Klotho was also significantly reduced (*P* < 0.01, Fig. 2b**/c**, Supplementary Figure 1). For the Wnt/β-catenin signaling pathway, cyclin D1 was mainly expressed in the nucleus whereas the two other important targets (β-catenin and c-Myc) were expressed in both the cytoplasm and the nucleus (Fig. 2d, Supplementary Figure 2). In the cytoplasm, compared with the control group, the protein level of β-catenin was significantly increased in the model group (*P* < 0.05, Fig. 2e). In the nucleus, compared with the control group, the protein levels of β-catenin, Cyclin D1 and c-Myc were also significantly increased (*P* < 0.01, Fig. 2f). Furthermore, as shown in the results of immunofluorescence staining, the expression level of β-catenin in the model cell was significantly higher than that in the control group (Fig. 2g). All of these results showed that the process of foam cell formation is closely related to the decrease in Klotho expression and the activation of the Wnt/β-catenin signaling pathway.

### Effect of recombinant Klotho protein on the viability of THP-1 macrophage-derived foam cells

**Fig. 3:**
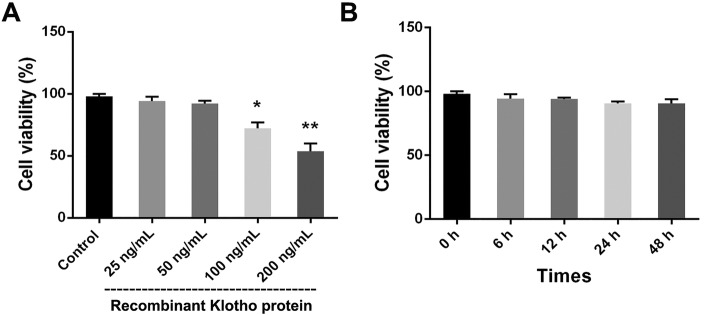
Effect of different concentrations of recombinant Klotho on cell viability of foam cells. **a** Foam cells were incubated with various concentrations of recombinant Klotho protein (0 ng/mL, 25 ng/mL, 50 ng/mL, 100 ng/mL, and 200 ng/mL) for 24 h. **b** Foam cells were incubated with 50 ng/mL recombinant Klotho for different periods of time (0 h, 6 h, 12 h, 24 h, and 48 h)d. ^*^P < 0.05 and ^**^P < 0.01 vs. control group

In this part, we detected the effect of recombinant Klotho on the viability of THP-1 macrophage-derived foam cells. After 4 h of synchronized growth, the medium of foam cells was replaced with fresh serum-free medium containing various recombinant Klotho at various concentrations (25 ng/mL, 50 ng/mL, 100 ng/mL, and 200 ng/mL) and the cells were then incubated for 24 h. The results of the MTT assay indicated that the viability of foam cells decreased with increasing concentrations of recombinant Klotho, with the most prominent effect observed at 200 ng/mL (*P* < 0.01). However, the cell viability was not altered at a low concentration of 50 ng/mL (Fig. 3a). Then, the foam cells were incubated with 50 ng/mL recombinant Klotho for 0 h, 6 h, 12 h, 24 h, and 48 h to investigate whether the recombinant Klotho reduces cell viability in a time-dependent manner. As shown in Fig. 3b, the viability of the foam cells was not altered when they were incubated with 50 ng/mL recombinant Klotho for different periods of time.

### Recombinant Klotho reduces lipid accumulation in THP-1 macrophage-derived foam cells

**Fig. 4:**
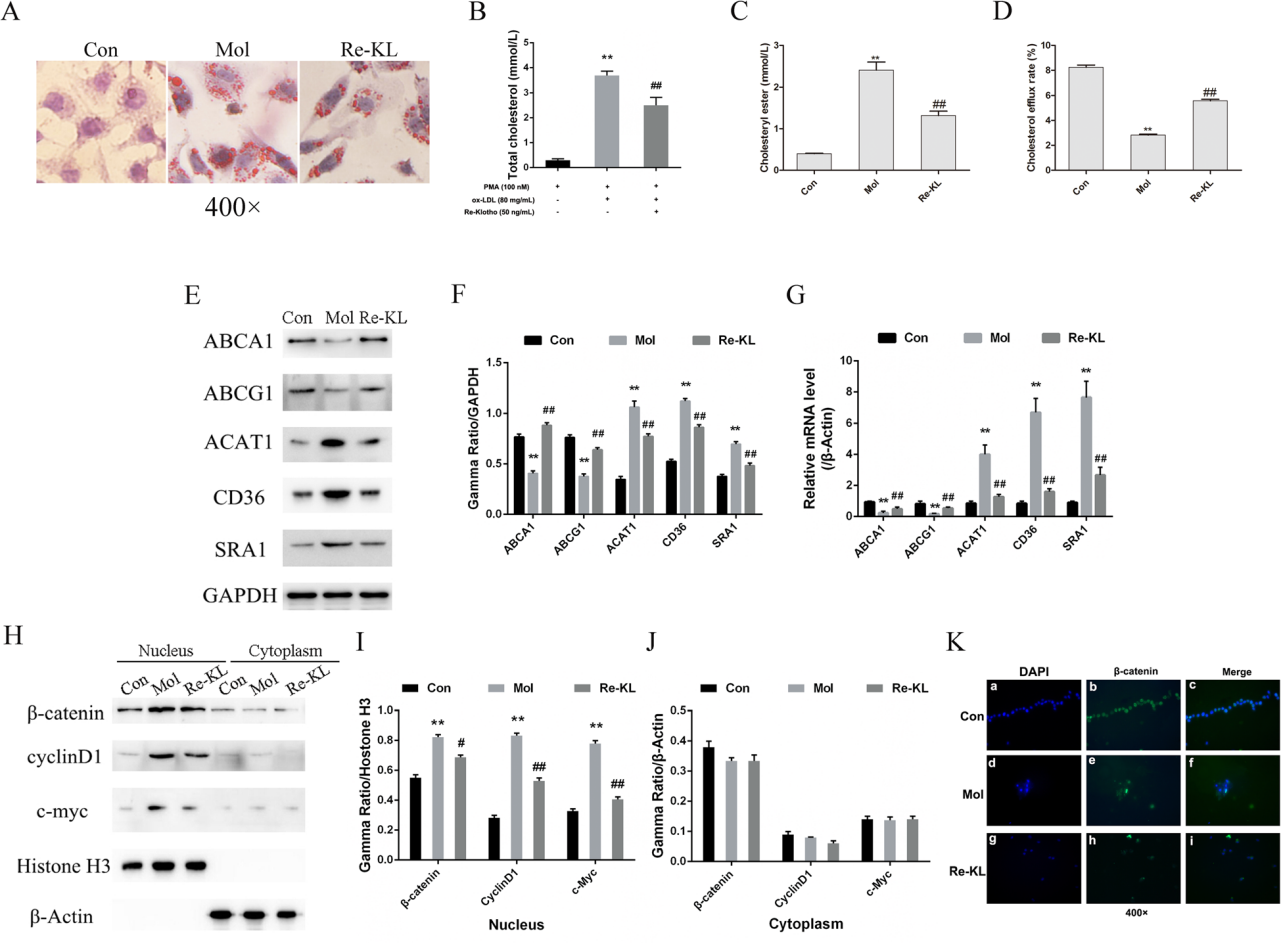
Effect of recombinant Klotho on cholesterol metabolism in foam cells. **a** Oil red O staining (400×); **b** Total cholesterol level assay; **c** Cholesteryl ester assay; **d** Cholesterol efflux rate assay; **e/f** Protein level expression of cholesterol metabolism related proteins (ABCA1, ABCG1, ACAT1, CD36 and SRA1); **g** mRNA level expression of cholesterol metabolism related genes (ABCA1, ABCG1, ACAT1, CD36 and SRA1); **h/i/j** Expression of key molecules related to Wnt/β-catenin signaling pathway (β-catenin, Cyclin D1 and c-Myc); **k** Immunofluorescence staining of β-catenin (400×); **a/d/g** DAPI staining (blue); **b/e/h** β-catenin staining (green); **c/f/i** Merge; Con, Control group; Mol, Model group; Re-Klotho, recombinant Klotho group; PMA, phorbol myristate acetate; ox-LDL, oxidized low-density lipoprotein. ^**^P < 0.01 vs. control group; ^#^*P* < 0.05 and ^##^*P* < 0.01 vs. model group

The THP-1 macrophage-derived foam cells were incubated with 50 ng/mL recombinant Klotho for 24 h to investigate whether the recombinant Klotho reduces lipid accumulation. As shown in the results of oil red O staining, compared with the model group, recombinant Klotho at 50 ng/mL significantly decreased intracellular lipid droplet accumulation (Fig. 4a). Similar to oil red O staining, biochemical testing also showed the lipid-lowering effect of recombinant Klotho in foam cells (*P* < 0.01; Fig. 4b). The results of cholesterol ester test showed that the content of cholesterol ester in Re-KL group was significantly lower than that in model group (*P* < 0.01; Fig. 4c). In order to observed the effect of Klotho protein on cholesterol metabolism, the cholesterol efflux rate of cells was detected. The results showed that compared with the control, the cholesterol efflux rate of the model group was significantly reduced, the results were reversed when treated with Klotho protein (*P* < 0.01; Fig. 4d). As shown in cholesterol metabolism related genes testing results (Fig. 4e/f/g, Supplementary Figure 3), compared with the model group, 50 ng/mL recombinant Klotho significantly increased the expression of RCT-related indicators (ABCA1 and ABCG1) (*P* < 0.01). However, the expression of cholesterol intake-related indicators (ACAT1, CD36 and SR-A1) was significantly reduced with the induction of recombinant Klotho (*P* < 0.01). In addition, in terms of the Wnt/β-catenin signaling pathway, compared with the model group, the nuclear protein levels of β-catenin, Cyclin D1 and c-Myc in foam cells were significantly decreased by the stimulus of recombinant Klotho (*P* < 0.01, Fig. 4h/i**,** Supplementary Figure 4). Nonetheless, there was no significant change in the cytoplasmic protein levels of β-catenin, Cyclin D1 and c-Myc between the groups (Fig. 4j). Importantly, as shown in the results of immunofluorescence staining, the expression level of β-catenin in the Re-KL group cell was significantly lower than that in the model group (Fig. 4k). All of these results showed that recombinant Klotho could effectively reduce lipid accumulation in THP-1 macrophage-derived foam cells and promote the occurrence and development of the RCT process. These phenomena may be closely related to the inhibitory Wnt/β-catenin signaling pathway in the induction of recombinant Klotho.

### Wnt/β-catenin signaling pathway participates in the recombinant Klotho -induced cholesterol efflux in THP-1 macrophage-derived foam cells

**Fig. 5:**
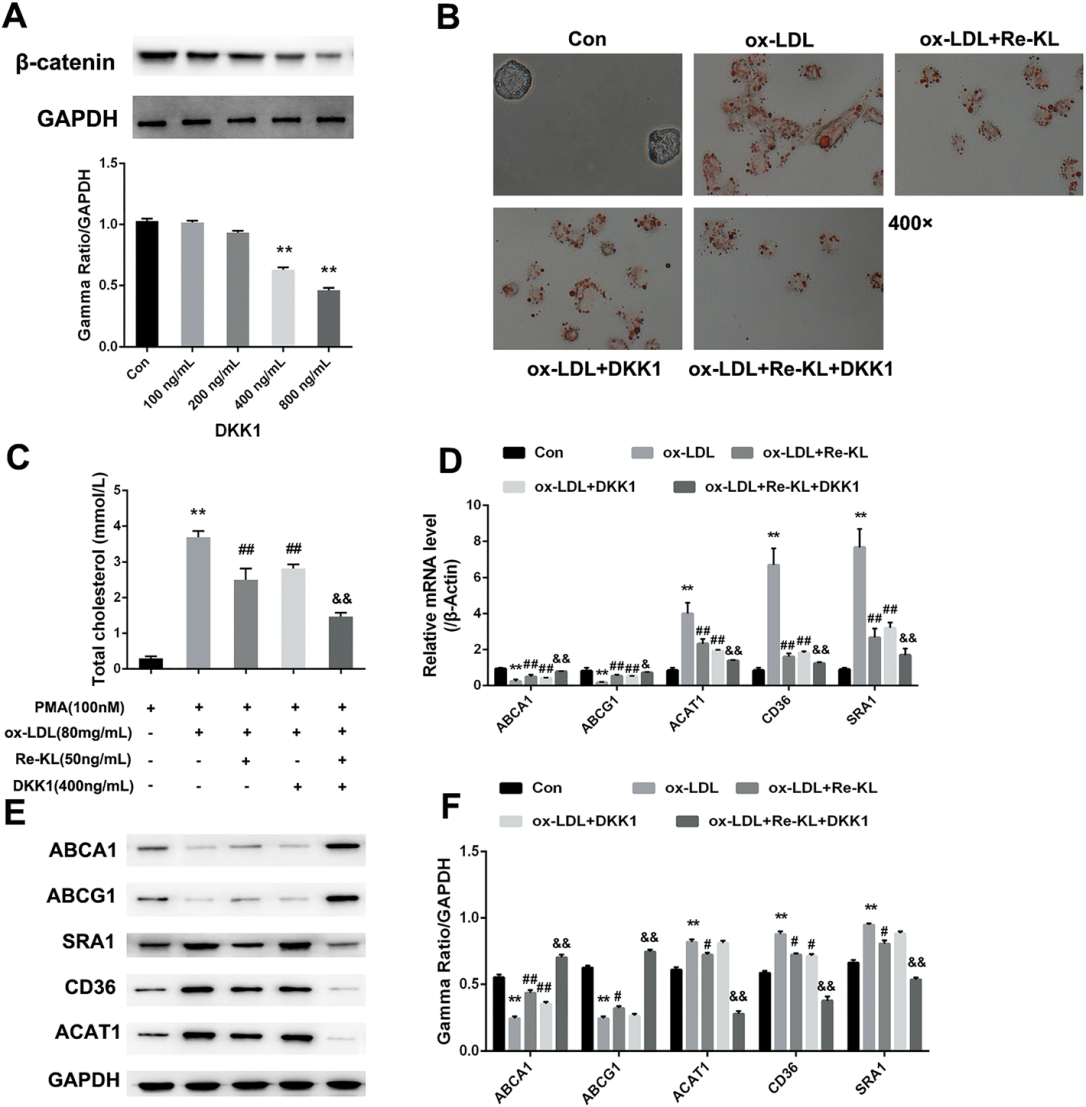
Effect of recombinant Klotho on Wnt/β-catenin signaling pathway. **a** Screening of the optimal concentration of foam cells by Wnt/β-catenin signaling pathway inhibitor DKK1. **b** Oil red O staining (400×); **c** Total cholesterol level assay; **d** mRNA level expression of cholesterol metabolism-related genes (ABCA1, ABCG1, ACAT1, CD36 and SRA1); **e/f** Expression of cholesterol metabolism related proteins (ABCA1, ABCG1, ACAT1, CD36 and SRA1). Con, Control group; Re-KL, recombinant Klotho group; PMA, phorbol myristate acetate; ox-LDL, oxidized low-density lipoprotein; DKK1, dickkopf-related protein 1. ^**^P < 0.01 vs. control group; ^#^*P* < 0.05 and ^##^*P* < 0.01 vs. model (ox-LDL) group; ^&^*P* < 0.05 and ^&&^*P* < 0.01 vs. DKK1 (ox-LDL + DKK1) group

To investigate the correlation between the role of recombinant Klotho in foam cells and the Wnt/β-catenin signaling pathway, we selected DKK1, an inhibitor of the Wnt/β-catenin signaling pathway, for joint research. As shown in Western blot testing result, 400 ng/mL DKK1 significantly inhibited the expression of β-catenin and was selected as the acting concentration for foam cells in subsequent studies (*P* < 0.01, Fig. 5a, Supplementary Figure 5). As the results shown in oil red O staining, compared with model group, the amount of cumulative red lipids was significantly reduced in the Re-KL and DKK1 treatment groups. Compared with the effect of DKK1 alone, the combined effect of Re-KL and DKK1 was more prominent (Fig. 5b). Similar to the results of oil red O staining, biochemical testing also illustrated this phenomenon (Fig. 5c). In addition, as shown in the evaluation of genes related to cholesterol metabolism (Fig. 5d/e/f, Supplementary Figure 6), compared with the model group, the expression levels of ABCA1 and ABCG1 were significantly increased in the Re-KL and DKK1 treatment groups (*P* < 0.05, *P* < 0.01). Meanwhile, the expression levels of ABCA1 and ABCG1 were also significantly increased in the combined treatment group compared with those in single DKK1 group (*P* < 0.05, *P* < 0.01). However, compared with ABCA1 and ABCG1, the expression levels of cholesterol intake-related indicators (ACAT1, CD36 and SR-A1) showed the opposite trend (*P* < 0.05, *P* < 0.01). These results indicated that recombinant Klotho protein may play a similar role as the Wnt/β-catenin signaling pathway inhibitor DKK1 on the regulation of THP-1 macrophage-derived foam cells.

## Discussion

In recent years, researchers have devoted increasing attention to the development of drugs against cardiovascular diseases, mainly through fat reduction, anti-inflammatory mechanisms, and other aspects. Atherosclerosis (AS) is the basis of cardiovascular diseases, accompanied by cellular cholesterol accumulates in lipid-engorged macrophage foam cells, thereby driving lipid deposition to the core of AS [24]. Thus, inhibiting the formation of foam cells is an important therapeutic strategy in AS treatment. A number of previous studies have shown that sustained uptake of cholesterol by macrophages depends largely on the active function of related proteins, such as the members of the scavenger family (SR-A1 and CD36) and regulatory protein (ACAT1) for cholesterol metabolism [25]. Oppositely, ABCA1 and ABCG1 mediate cholesterol efflux and play important roles in the RCT process [10, 12, 26]. A study has clearly demonstrated the anti-inflammatory effect of the Klotho on AS [20], but its lipid-lowering effect is still rarely reported. In this study, we found that the expression of Klotho significantly decreased in a THP-1 macrophage-derived foam cell model, suggesting that Klotho can regulate cholesterol uptake by macrophages. Finally, our data demonstrated that recombinant Klotho up-regulated the expression of ABCA1 and ABCG1 and decreased the expression of cholesterol intake-related indicators (ACAT1, CD36 and SR-A1) to reduce cellular cholesterol accumulation by inhibiting the Wnt/β-catenin signaling pathway in THP-1 macrophage-derived foam cells.

A recent study has reported that myocardial biopsies from patients with a high risk of 10-year atherosclerotic cardiovascular disease (ASCVD) showed a significant reduction in Klotho expression as compared with that in cardiomyocytes from patients with low risk of ASCVD [27]. An additional study has also shown that patients with clinical atherosclerotic vascular disease have lower serum levels of Klotho [18]. Soluble Klotho has been proved to have direct protective effects on the vasculature, mainly by inhibiting smooth muscle cell calcification, suppressing the expression of adhesion molecules, resisting oxidative stress, and maintaining endothelial integrity and function [28]. In terms of clinical coronary disease, patients had lower soluble serum Klotho levels, which indicated a significant negative correlation with the severity of coronary stenosis [28, 29]. Previous experimental studies in animal models have shown that Klotho deficiency causes disruptions in endothelial homeostasis, further leading to endothelial dysfunction, which is a critical factor in the pathogenesis of AS [30, 31]. Importantly, it has been proved that these alterations may be reversed by administering the Klotho gene, laying a solid foundation for the cell experiments in our study.

AS is generally recognized as a chronic inflammatory disorder of the artery wall, but inflammation is mainly triggered by risk factors such as elevated plasma cholesterol levels and hypertension [32]. In particular, higher levels of total cholesterol and the accumulation of macrophages were considered as powerful killers of AS [33]. Therefore, it is extremely important to ensure the normal metabolism of cholesterol. RCT refers to the transport of cholesterol in peripheral tissues that cannot regulate and utilize cholesterol to the liver and is utilized in sterol-producing tissues, which may alleviate or even reverse the already formed atherosclerotic lipid plaques through cholesterol reverse transport. The process mainly includes four steps: cholesterol efflux from peripheral tissue, cholesterol esterification in HDL granules, cholesterol ester transfer in HDL and HDL clearance [10, 34, 35]. It is believed that the RCT, a process that removes excess cholesterol from peripheral tissues/cells including macrophages to circulating HDL, is one of the main mechanisms responsible for the anti-atherogenic properties of HDL [36]. ATP-binding cassette transporter A1 (ABCA1) plays a key role in RCT. ABCA1 uses ATP as energy source to transport excess cholesterol and phospholipids from cells to primary HDL and inhibit their conversion in foam cells, which is the first key part of RCT [26, 37]. Our study shows for the first time the low expression of Klotho at the mRNA and protein levels in the transformation of macrophages into foam cells, which was accompanied by the accumulation of intracellular cholesterol. In addition, exogenous recombinant Klotho significantly promoted the RCT process in THP-1 macrophage-derived foam cells, as evidenced by the reduced cholesterol accumulation and increased expression levels of ABCA1 and ABCG1. To further study whether the effect of recommended Klotho induced choice efflux is related to Wnt/β-catenin pathway, we choose DKK1, an initiator of the Wnt/β-catenin pathway in the following experiments.

Increasing evidence has suggested that the Wnt/β-catenin signaling pathway is an important molecular mechanism for cardiovascular diseases [38]. In early atherosclerosis in obesity, the Wnt/β-catenin signaling pathway inhibitor sclerostin could serve as a useful biomarker [39]. The possible mechanism of selenium deficiency-induced cardiac dysfunction was also proved to be associated with the Wnt/β-catenin signaling pathway [40]. In the study of a diabetic myocardial infarction model, the regulatory role of thioredoxin-interacting protein on the Wnt/β-catenin signaling pathway was mainly achieved by altering the levels of reactive oxygen species, which was accompanied by changes in the expression of its downstream proteins cyclin D1 and C-myc [41]. Related research on cardiogenesis and lineage diversification of the heart has shown that the Wnt/β-catenin pathway is a major component by which cardiac mesenchymal cells modulate the pre-specification, renewal, and differentiation of Isl1+ cardiovascular progenitors [42]. Aging is the main risk factor for cardiovascular diseases, and its mechanism has been shown to be related to Wnt/β-catenin pathway signal activation, which is mainly manifested in the increased β-catenin-activated phosphorylation at position Ser675 in aging mammary arteries [43]. As a classical pathogenic mechanism of a variety of cardiovascular diseases, stress-induced cardiomyocyte apoptosis has been proved to be related to the reduction of anti-apoptotic Bcl-2 and the increase in pro-apoptotic Bax and cleaved caspase-3 via activing the Wnt/β-catenin pathway [44]. Due to the universality of the Wnt/β-catenin signaling pathway in cardiovascular diseases, most disease therapies that target this pathway have achieved good results. In the ox-LDL-induced HUVEC injury model, the biomarkers of the Wnt/β-catenin pathway were activated, as mainly reflected by the up-regulation of β-catenin, Dvl-1 and Cyclin D1. Additionally, ox-LDL-induced HUVEC injury and apoptosis, oxidative stress, and activation of the Wnt/β-catenin pathway were suppressed by pigment epithelium-derived factor [45]. In the apolipoprotein E-deficient (ApoE61/61) mouse model, the administration of recombinant mouse sclerostin protein, an inhibitor of the Wnt signaling pathway, inhibited angiotensin II-induced atherosclerosis [46]. In our study, the Wnt/β-catenin pathway was activated in the formation of foam cells. Interestingly, similar to DKK1 (Wnt/β-catenin signaling pathway inhibitor), recombinant Klotho significantly reduced the activity of the Wnt/β-catenin pathway, which was accompanied by the down-regulation of β-catenin, Dvl-1, and cyclin D1, the enhancement of cholesterol efflux capacity, and changes in the expression levels of cholesterol metabolism-related proteins.

## Conclusions

Study proves that recombinant Klotho reduces lipid accumulation through the regulation of cholesterol metabolism-related proteins via inhibiting the Wnt/β-catenin pathway in THP-1 macrophage-derived foam cells. Treatment with recombinant Klotho in the field of cardiovascular diseases and the research and development of drugs that regulate signaling pathways may provide novel strategies for the prevention and treatment of atherosclerosis. Undoubtedly, these findings offer a new perspective for the treatment of atherosclerosis.

## Supplementary information

**Additional file 1: Figure S1.** Unprocessed original scans for the blots.

**Additional file 2: Figure S2.** Unprocessed original scans for the blots.

**Additional file 3: Figure S3.** Unprocessed original scans for the blots.

**Additional file 4: Figure S4.** Unprocessed original scans for the blots.

**Additional file 5: Figure S5.** Unprocessed original scans for the blots.

**Additional file 6: Figure S6.** Unprocessed original scans for the blots.

## Data Availability

The datasets used and/or analysed during the current study are available from the corresponding author on reasonable request.

## Abbreviations

ABCA1: ATP-binding cassette transporter A1
ACAT: Acyl coenzyme a-cholesterol acyltransferase
AS: Atherosclerosis
ASCVD: Atherosclerotic cardiovascular disease
HDL: High density lipoprotein
HUVEC: Human umbilical vein endothelial cells
ox-LDL: Oxidized low-density lipoprotein
qRT-PCR: Quantitative reverse-transcription polymerase chain reaction
RCT: Reverse cholesterol transport
Re-KL: Recombinant Klotho
SR-A1: Scavenger receptor-A1
