# Supplementary figures and images for: Recombinant Klotho protein enhances cholesterol efflux of THP-1 macrophage-derived foam cells via suppressing Wnt/β-catenin signaling pathway
Source: BMC Cardiovasc Disord. 2020 Mar 5;20:120. doi: 10.1186/s12872-020-01400-9 (PMC7059691; doi:10.1186/s12872-020-01400-9)

KL:

Control Model


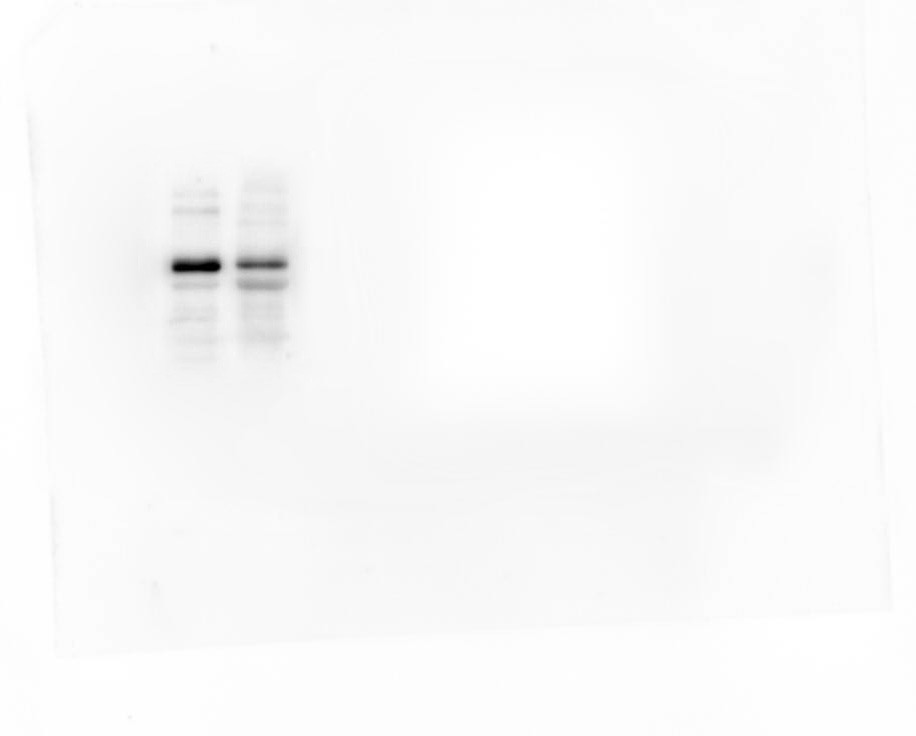


GAPDH:

Control Model


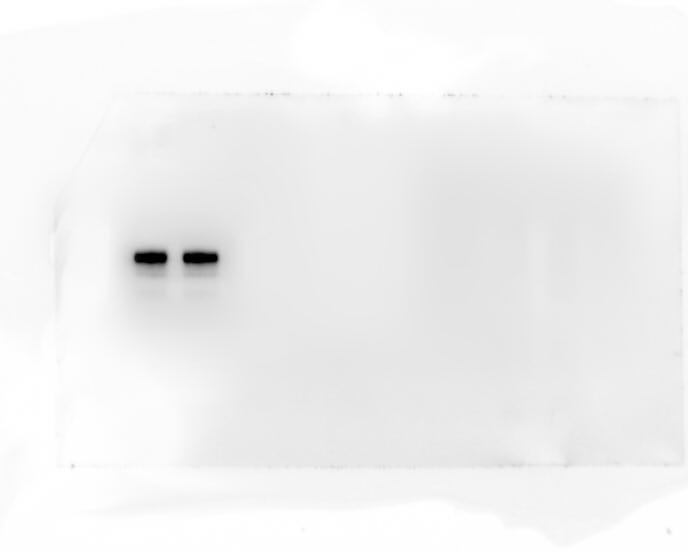

Supplement: Supplementary file 1 — Additional file 1: Figure S1. Unprocessed original scans for the blots. [file 12872_2020_1400_MOESM1_ESM.doc]

β-catenin:

Control 100 ng/ml 200ng/ml 400ng/ml 800ng/ml


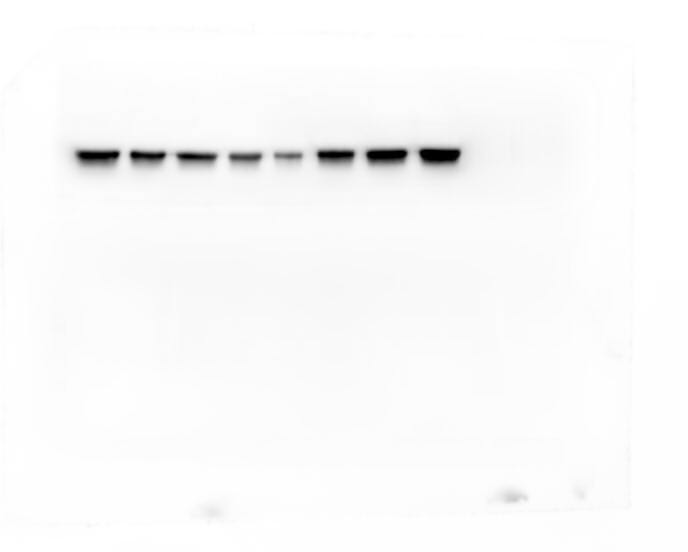


GAPDH:

Control 100 ng/ml 200ng/ml 400ng/ml 800ng/ml


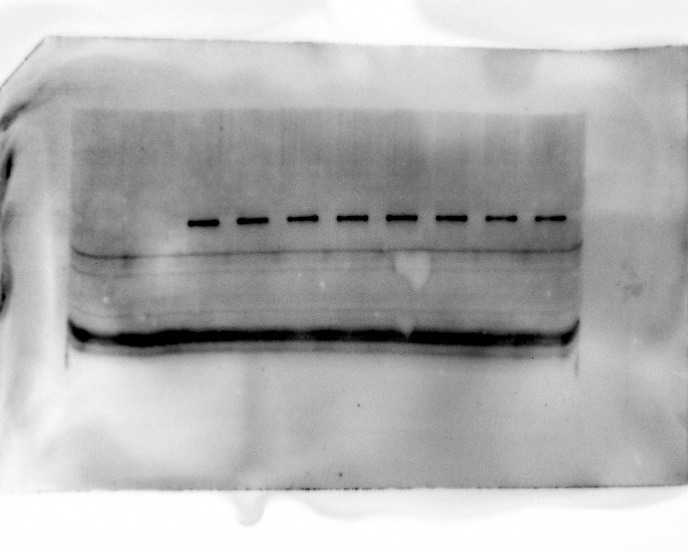

Supplement: Supplementary file 5 — Additional file 5: Figure S5. Unprocessed original scans for the blots. [file 12872_2020_1400_MOESM5_ESM.doc]

1: PMA (100 nM) 2: ox-LDL (80mg/ml) 3: Re-KL (50ng/ml) 4: DKK1 (400ng/ml)

ABCA1:

1 1+2 1+2+3 1+2+4 1+2+3+4


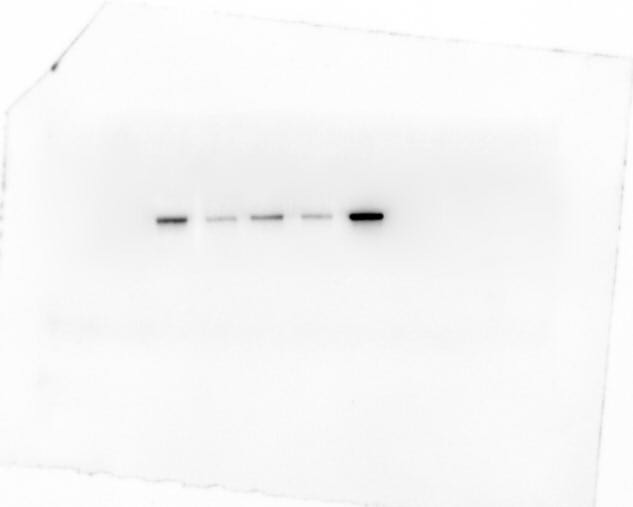


ABCG1:

1 1+2 1+2+3 1+2+4 1+2+3+4


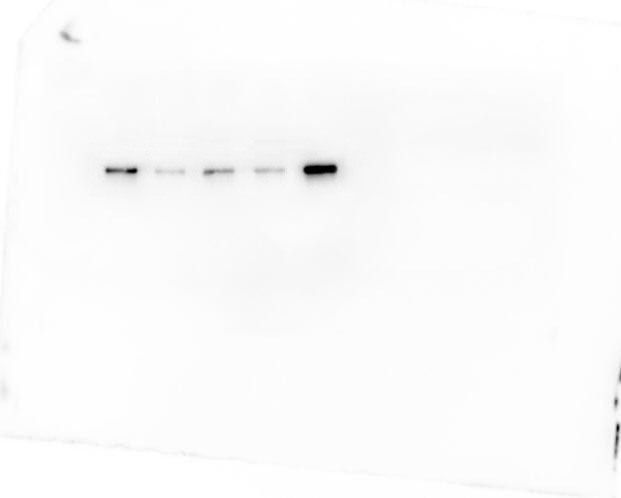


SRA1:

1 1+2 1+2+3 1+2+4 1+2+3+4


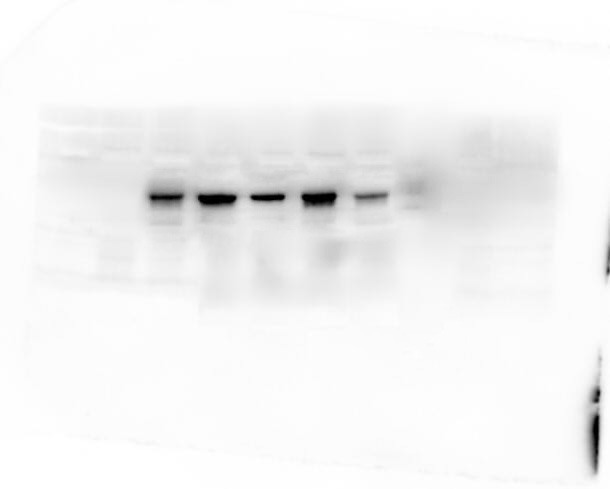


CD36:

1 1+2 1+2+3 1+2+4 1+2+3+4


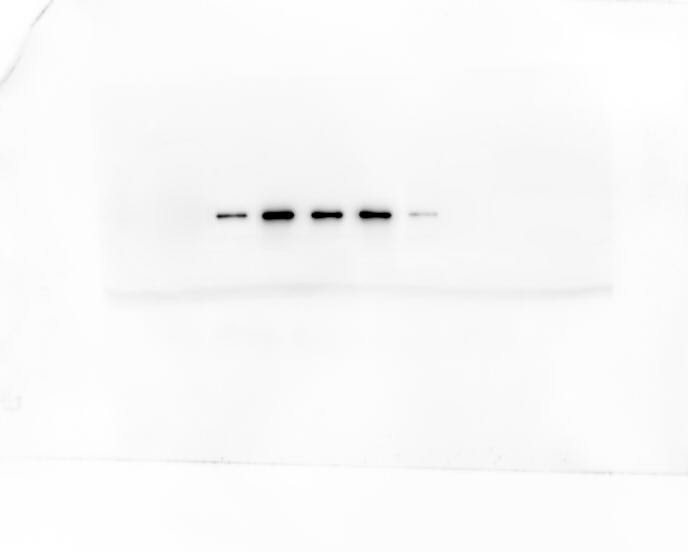


ACAT1:

1 1+2 1+2+3 1+2+4 1+2+3+4


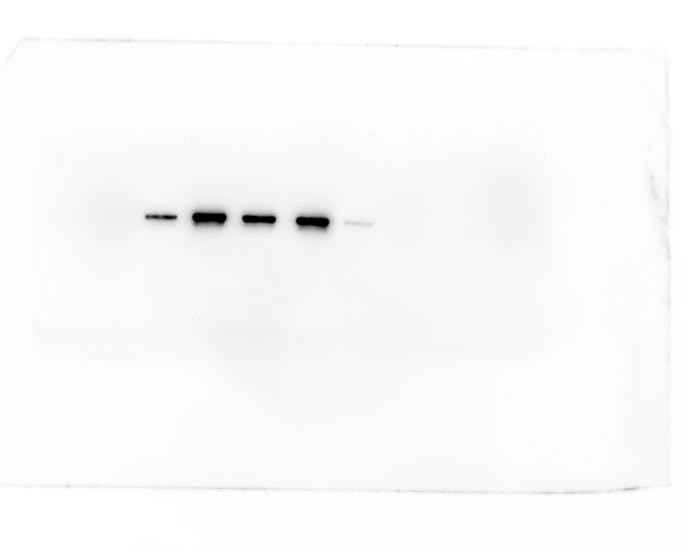


GAPDH:

1 1+2 1+2+3 1+2+4 1+2+3+4


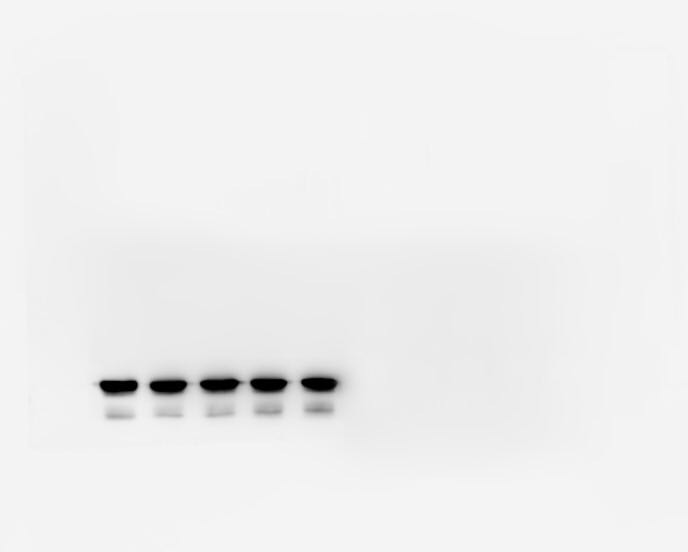

Supplement: Supplementary file 6 — Additional file 6: Figure S6. Unprocessed original scans for the blots. [file 12872_2020_1400_MOESM6_ESM.doc]
