# Supplementary material for: Recombinant Klotho protein enhances cholesterol efflux of THP-1 macrophage-derived foam cells via suppressing Wnt/β-catenin signaling pathway
Source: BMC Cardiovasc Disord. 2020 Mar 5;20:120. doi: 10.1186/s12872-020-01400-9 (PMC7059691; doi:10.1186/s12872-020-01400-9)

β-catenin：

Control Model Control Model

(cytoplasm) (cytoplasm) (nucleus) (nucleus)


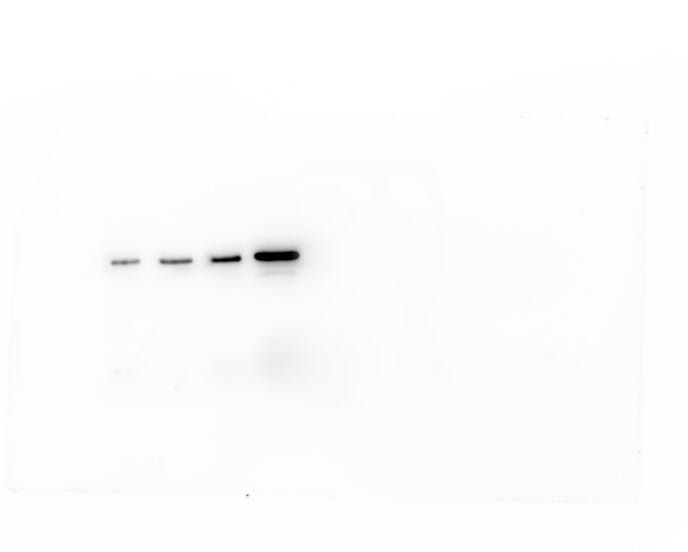


Cyclin D1:

Control Model Control Model

(cytoplasm) (cytoplasm) (nucleus) (nucleus)


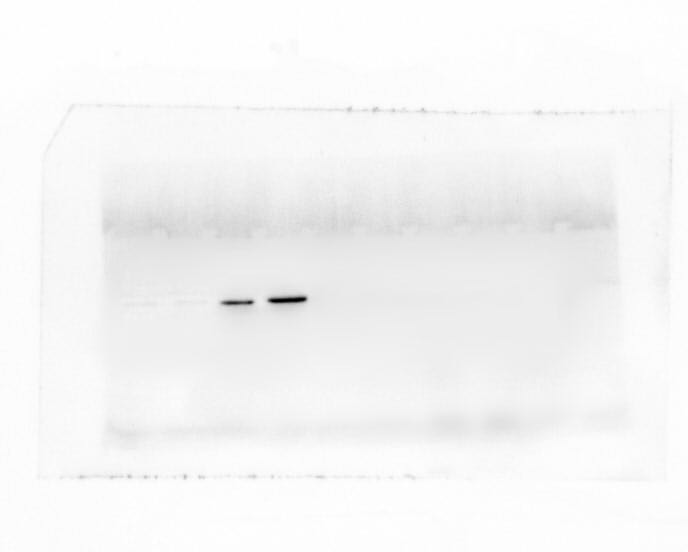


c-Myc:

Control Model Control Model

(cytoplasm) (cytoplasm) (nucleus) (nucleus)


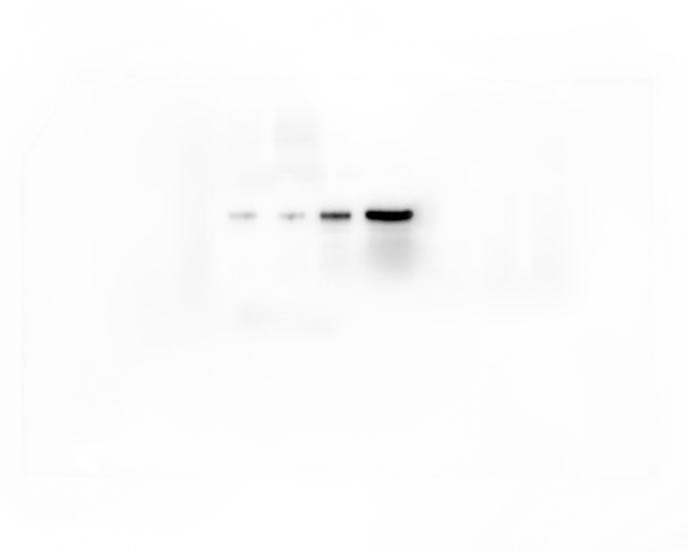


Histone H3:

Control Model Control Model

(cytoplasm) (cytoplasm) (nucleus) (nucleus)


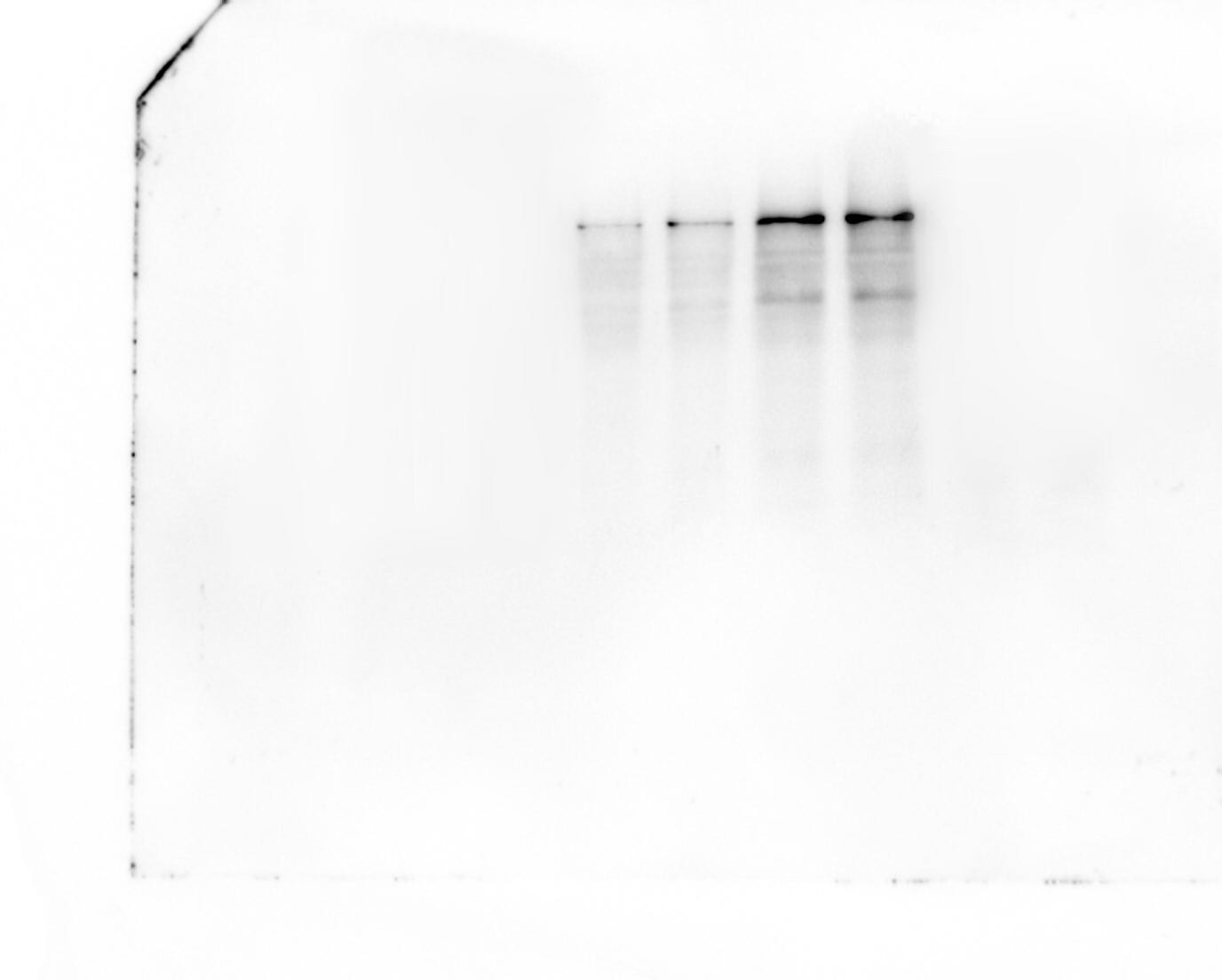


β-actin:

Control Model Control Model

(cytoplasm) (cytoplasm) (nucleus) (nucleus)


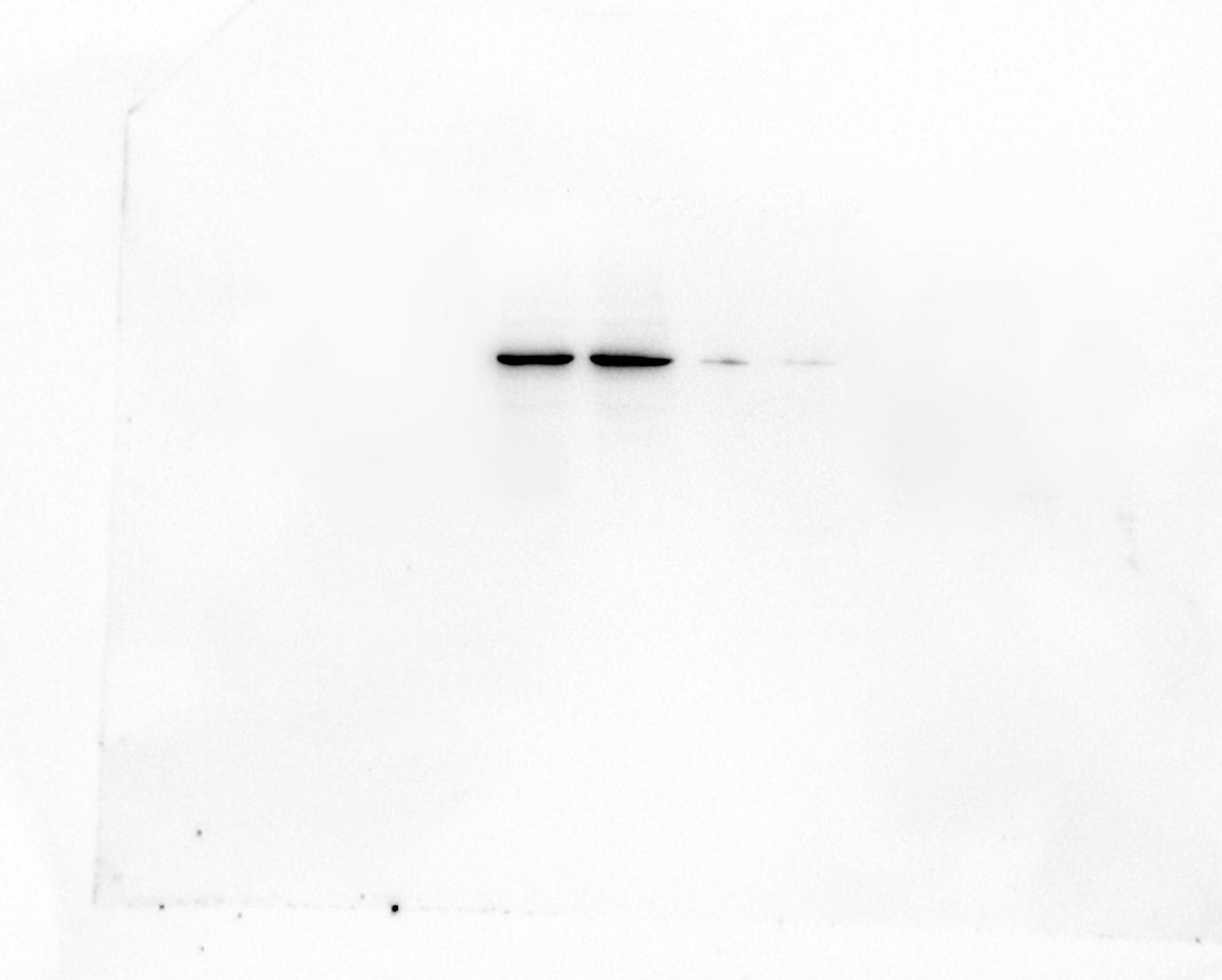

Supplement: Supplementary file 2 — Additional file 2: Figure S2. Unprocessed original scans for the blots. [file 12872_2020_1400_MOESM2_ESM.doc]
