# Supplementary material for: Recombinant Klotho protein enhances cholesterol efflux of THP-1 macrophage-derived foam cells via suppressing Wnt/β-catenin signaling pathway
Source: BMC Cardiovasc Disord. 2020 Mar 5;20:120. doi: 10.1186/s12872-020-01400-9 (PMC7059691; doi:10.1186/s12872-020-01400-9)

ABCA1:

Control Model Re-KL


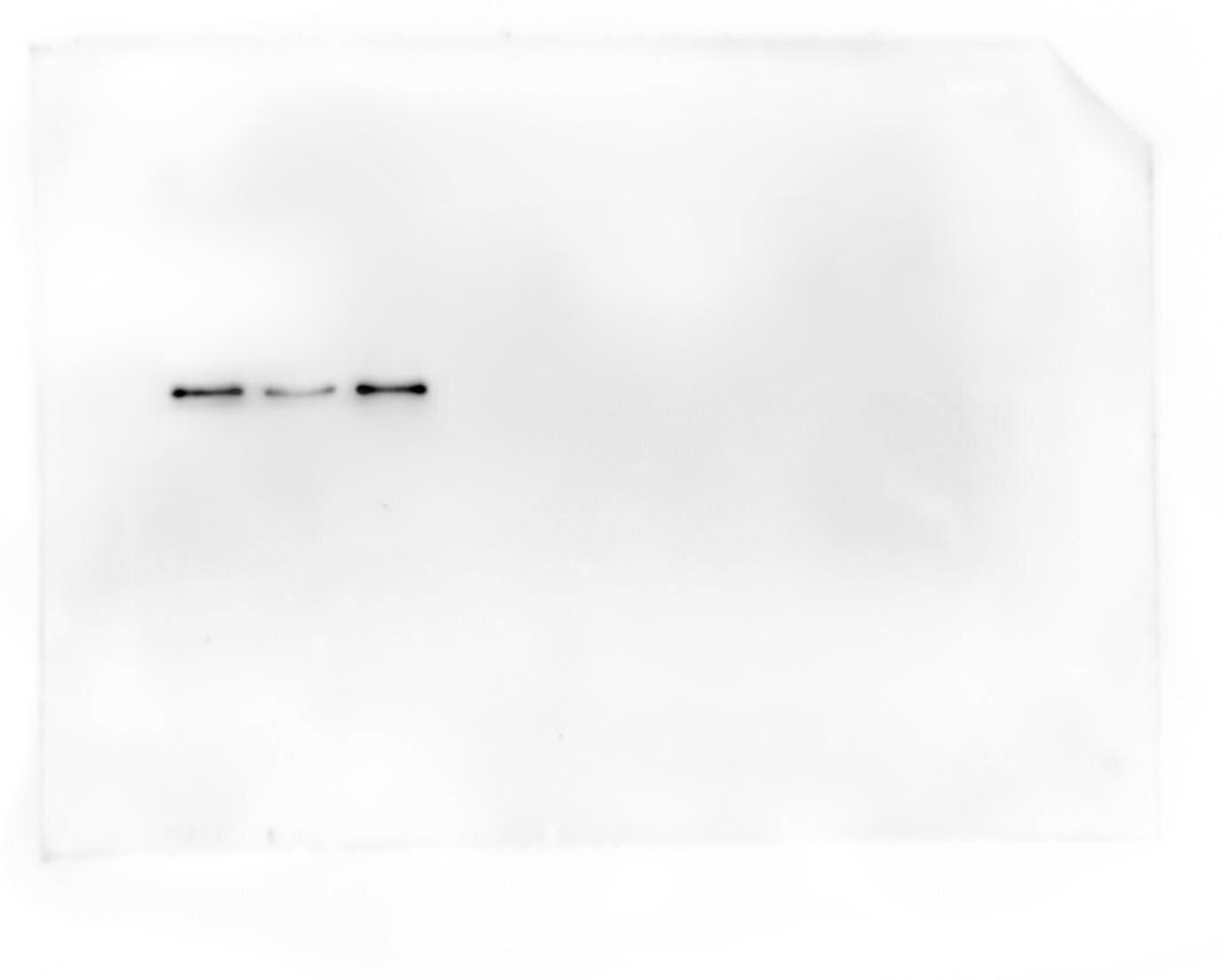


ABCG1:

Control Model Re-KL


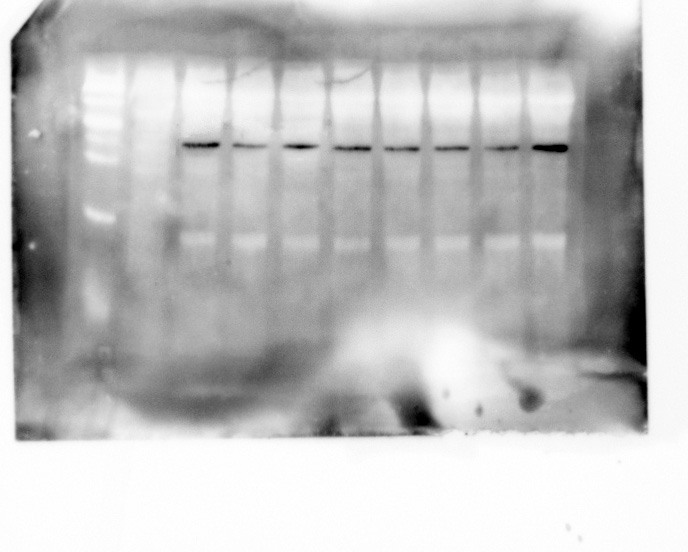


ACAT1:

Control Model Re-KL


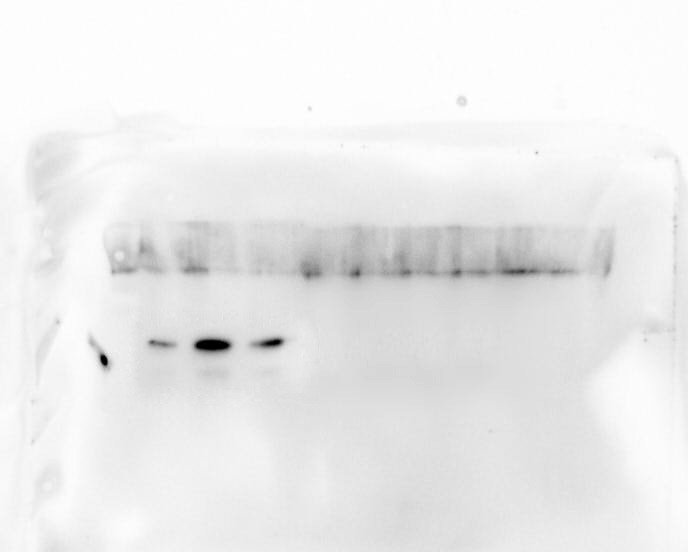


CD36:

Control Model Re-KL


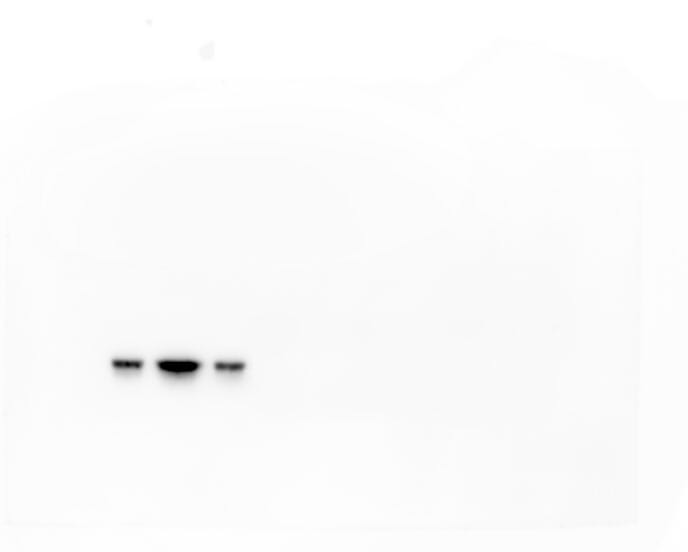


SRA1:

Control Model Re-KL


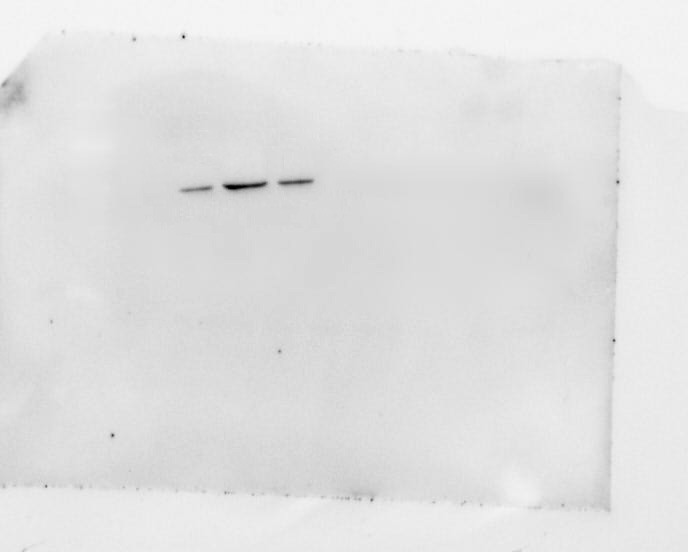


GAPDH:

Control Model Re-KL


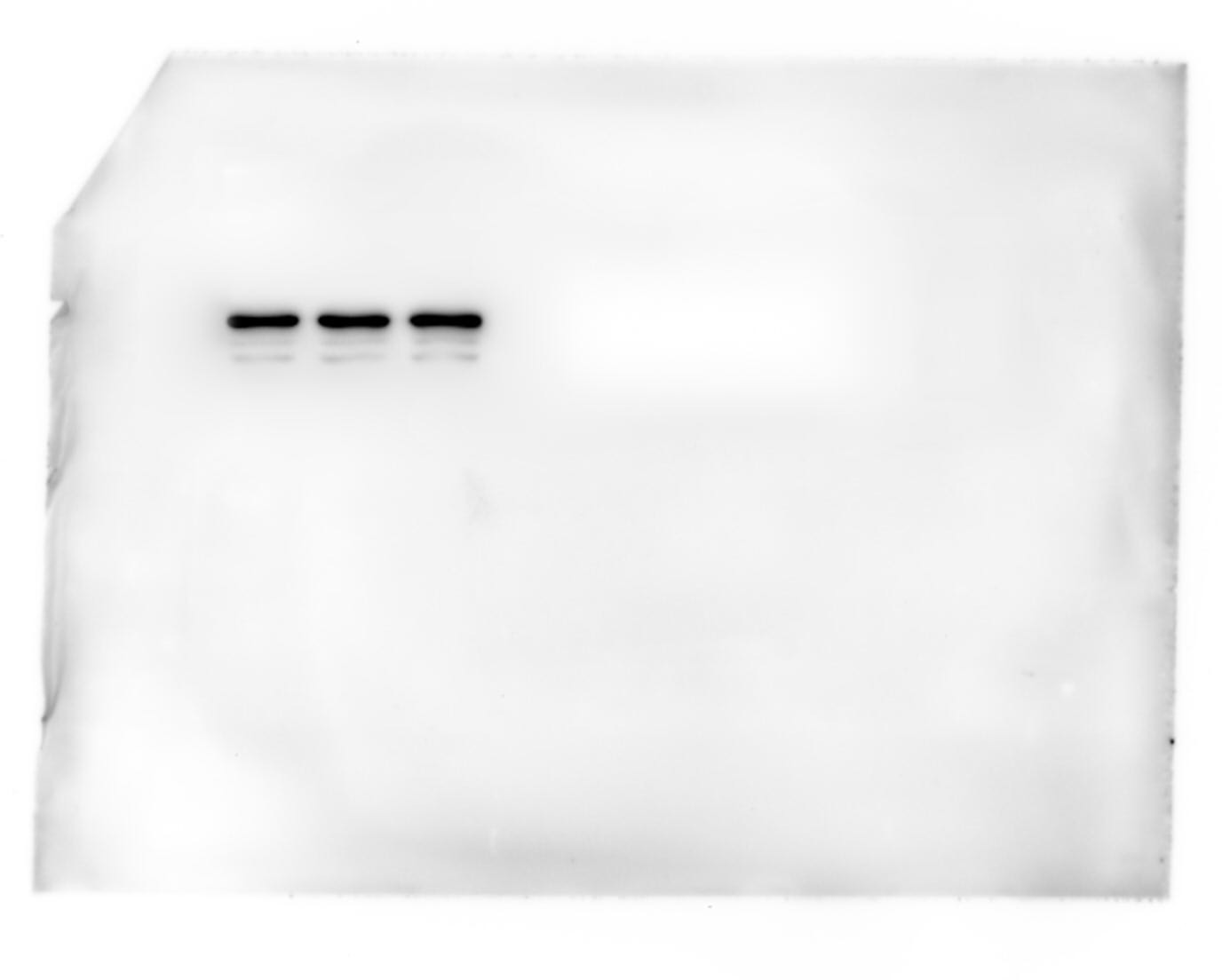

Supplement: Supplementary file 3 — Additional file 3: Figure S3. Unprocessed original scans for the blots. [file 12872_2020_1400_MOESM3_ESM.doc]
