# Supplementary material for: Recombinant Klotho protein enhances cholesterol efflux of THP-1 macrophage-derived foam cells via suppressing Wnt/β-catenin signaling pathway
Source: BMC Cardiovasc Disord. 2020 Mar 5;20:120. doi: 10.1186/s12872-020-01400-9 (PMC7059691; doi:10.1186/s12872-020-01400-9)

β-catenin:

Control Model Re-KL Control Model Re-KL

(nucleus) (nucleus) (nucleus) (cytoplasm) (cytoplasm) (cytoplasm)


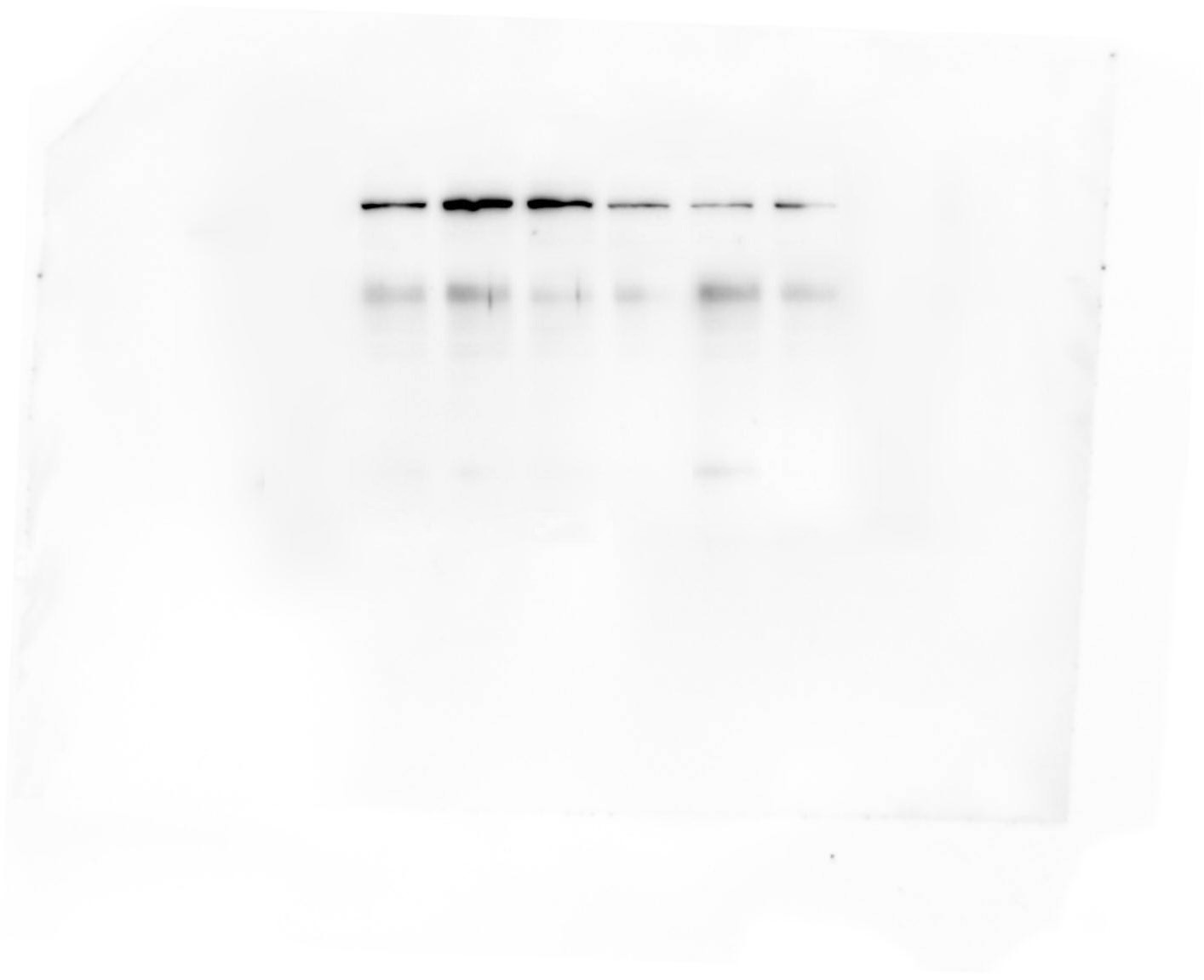


Cyclin D1:

Control Model Re-KL Control Model Re-KL

(nucleus) (nucleus) (nucleus) (cytoplasm) (cytoplasm) (cytoplasm)


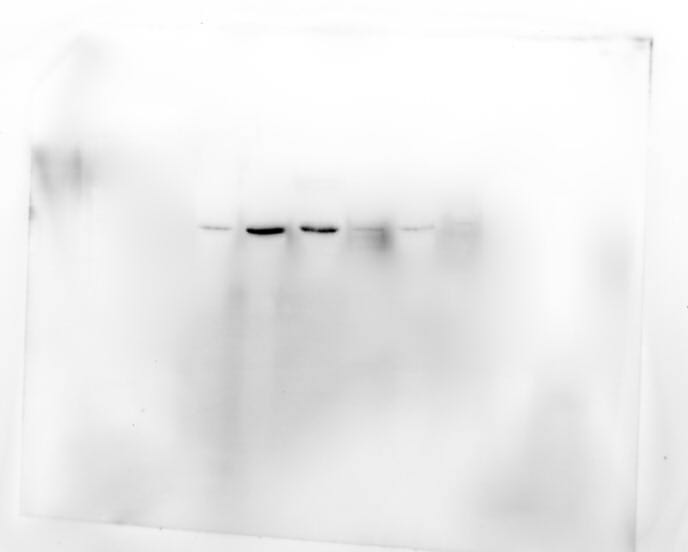


c-Myc:

Control Model Re-KL Control Model Re-KL

(nucleus) (nucleus) (nucleus) (cytoplasm) (cytoplasm) (cytoplasm)


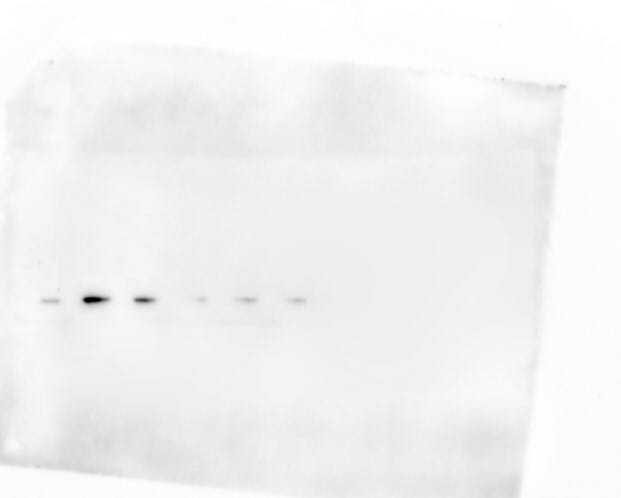


Histone H3:

Control Model Re-KL Control Model Re-KL

(nucleus) (nucleus) (nucleus) (cytoplasm) (cytoplasm) (cytoplasm)


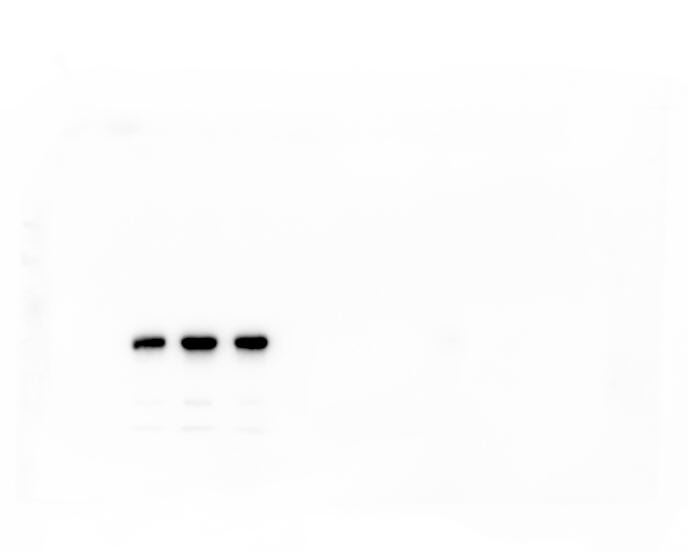


β-actin

Control Model Re-KL Control Model Re-KL

(nucleus) (nucleus) (nucleus) (cytoplasm) (cytoplasm) (cytoplasm)


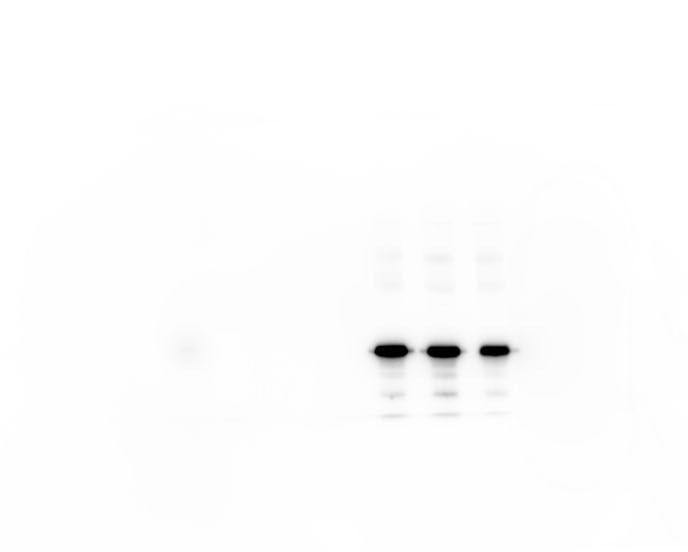

Supplement: Supplementary file 4 — Additional file 4: Figure S4. Unprocessed original scans for the blots. [file 12872_2020_1400_MOESM4_ESM.doc]
